# Adolescent dental arch development among Southern Chinese in Hong Kong: a geometric morphometric approach

**DOI:** 10.1038/s41598-019-55073-2

**Published:** 2019-12-06

**Authors:** Yi Feng Wen, Hai Ming Wong, Tao Pei, Colman McGrath

**Affiliations:** 1 Key Laboratory of Shaanxi Province for Craniofacial Precision Medicine Research, College of Stomatology, Xi’an Jiaotong University, Xi’an, China; 2 Paediatric Dentistry, Faculty of Dentistry, The University of Hong Kong, Hong Kong, Hong Kong; 3 Peking University Shenzhen Hospital, Shenzhen, China; 4 Dental Public Health, Faculty of Dentistry, The University of Hong Kong, Hong Kong, Hong Kong

**Keywords:** Oral anatomy, Paediatric research

## Abstract

This study aimed to investigate changes in types of dental arch form during adolescence and explore adolescent changes in size and form of dental arch. Hong Kong Chinese were recruited and digital dental arch models were obtained at ages 12, 15, and 18 years. Geometric morphometrics was used to investigate adolescent changes of dental arch form. There were 225 participants from whom digital models at all three age periods were available. Three types of dental arch form were identified through clustering. Significant changes (p < 0.001) in types of dental arch form were noted during age 12–18 years. During age 12–18 years, significant changes in centroid size and form of dental arch were observed (p < 0.001). No significant changes were observed during 15–18 years. Adolescent changes of dental arch form occur primarily during age 12–15 years, whereas dental arch form was relatively stable during age 15–18 years.

## Introduction

Adolescence is a critical period during which dramatic physical and psychological development occurs. In the biological context, it has been defined as the physical transformations that occur from the onset of puberty to termination of growth^1^. In the dental context, adolescence spans from the age of 12 (±1 year) to 18 (±1 year) years, a period that starts from the end of mixed dentition/beginning of the permanent dentition and ends when maturation of dentition is achieved surrounding the beginning of adulthood.

Adolescence is a crucial period in orthodontics and craniofacial orthopedics, during which the mainstream interventions of orthodontics and pre-surgery orthodontic treatment in patients with skeletal deformities are performed. Decision making in clinical practice needs to take adolescent growth potential of the craniofacial complex into consideration. For some patients, puberty growth may further compromise existing malocclusion and needs to be compensated for during orthodontic treatment. In other cases, however, growth and development may partially correct for malocclusion. In-depth knowledge on growth and development of dental arch during adolescence is therefore of distinct clinical significance.

A large volume of literature has investigated dimensional changes in dental arch during adolescence. Most existing studies were devoted to growth changes in width^2,3^, depth^4,5^, length^4,6^ of the dental arch, tooth inclination^7,8^, and intra- and inter-arch discrepancy^9,10^. Findings from current studies have been inconsistent, which are commonly attributed to variations in study design, sample composition, age period under investigation, and methods of assessment. However, an additional limitation inherent in these studies is their use of a sparse set of parameters to characterize dental arch. Traditional measures of dental arch, such as width, length, and depth, cannot capture the complete geometric information stored in the complex 3-dimensional (3D) models of dental arch. These studies are therefore of limited power in identifying changes in dental arch during adolescence.

In contrast to traditional linear and angular measures of dental arch, geometric morphometrics is a revolutionarily new approach grounded on rigid statistical theory of shape^11^ to analyzing geometric characteristics of objects. It captures the complete geometric information contained by all landmarks^12^, thus increasing the power to detect changes in dental arch that are indiscernible using traditional methods, facilitating the discovery of robust changes in geometry of dental arch, and providing the opportunity to visualize adolescent changes of dental arch in 3D. In geometric morphometrics, size of an object is quantified by a composite measure termed centroid size, defined as the square root of sum of squared distance between each landmark and the centroid^13^. Shape, on the other hand, refers to the geometric properties of an object that remain after removal of the effect of location, scale, and rotation^13^. Geometric morphometrics has been used in analyzing shape of craniofacial complex^14^. Dental arch shape is of interest in fields as diverse as anthropology, oral and maxillofacial surgery, and orthodontics. Geometric morphometric analyses have been performed to investigate fluctuating asymmetry of dental arch among healthy population^15^ and dental arch shape of cleft^16^ and obstructive sleep apnoea^17^ patients. In orthodontics, dental arch characteristics of patients with orthodontic treatment need have been investigated^18,19^. In addition, Celar *et al*. explored the effect of different treatment modalities on shape changes of dental arch^20^.

In spite of the moderately sized literature, credibility of these studies is limited by their relatively small sample size and unrepresentative sampling methods. Furthermore, adolescent changes in shape of dental arch have not been studied longitudinally. Since both size and shape are of interest when considering adolescent changes of dental arch, form (combination of size and shape)^13^ of dental arch will be analyzed in this study. Based on a longitudinal sample of Southern Chinese in Hong Kong, the present study aimed to categorize dental arch form into clusters, to investigate changes in types of dental arch form during adolescence, and to explore adolescent changes in size and form of dental arch.

## Materials and Methods

### Study population

This was a prospective longitudinal study conducted among a population-representative sample of Southern Chinese in Hong Kong. Participants were excluded from current study if they (i) had mixed dentition; (ii) were undergoing or had received orthodontic treatment; (iii) had history of systematic diseases known to alter dental arch shape; (iv) had premature loss of at least three permanent teeth in either dental arch; (v) the quality of dental digital model was unacceptable; and (vi) had missing occlusal records/inaccurate occlusal recording.

Baseline of the study was performed when participants were aged 12 years. The sampling frame was all local secondary schools in Hong Kong. A random sample of 45 secondary schools from 18 districts in Hong Kong was selected as the primary sampling unit, approximating 10% of all local secondary schools. Within each school, all students in Form 1 and Form 2 classes (equivalent to Grade 6 and 7 in the United States) born between April 1 and May 31 were invited to participate in an oral health screening. Parents/primary caregivers provided written informed consent for their child to participate in the study and children provided their verbal ascent to participate. Two rounds of follow-ups were performed when participants were aged 15 years and 18 years, respectively. The study protocol was approved by the Institutional Review Board of the University of Hong Kong/Hospital Authority Hong Kong West Cluster (IRB reference number: UW 13–584 and UW15–278). We confirm that all methods were performed in accordance with the relevant guidelines and regulations.

### 3D digital dental models

Conventional upper and lower alginate dental impressions were obtained from participants. Impression-taking was conducted in a standardized manner with specific guidelines on tray selection, mixing and loading of alginate impressions, and the anatomical extent requirements of impressions^21^ by trained operators. Impressions were evaluated and repeat impressions were obtained if anatomical coverage was deficient. Impression were wrapped in wet gauze and transported in a pressure-free environment to the dental laboratory at the end of each assessment session. Conventional plaster models were obtained from alginate impressions and then these plaster models were scanned to provide 3D digital models by the Modern Dental Laboratory (http://www.moderndentallab.com/).

### Landmark digitization

For both maxillary and mandibular dentition, landmark digitization was performed for all teeth from bilateral central incisors to first permanent molars. All landmarks were digitized in accordance with the definition by Bishara *et al*.^6^, Thilander^22^, Heikinheimo *et al*.^10^. Clinical center point (the center point of the clinical axis of the tooth on the labial/buccal surface), mesial/distal point (the most prominent point on the mesial/distal surface of tooth), and buccal gingival points (the points on buccal or lingual gingival curvature that divide teeth into equal mesial and distal halves) were digitized for all teeth irrespective of the tooth type. In addition, incisal point (the central point in incisal edge of incisor) was digitized for central and lateral incisors, cusp point (tip point of tooth cusp) was digitized for canine, and occlusal point (the central pit in central fossa or the point where the buccal groove cross buccal marginal ridge into central fossa when central pit is ambiguous for molars and midpoint between mesial fossa and distal fossa for premolars) was digitized for premolars and molars. Digitization was performed using a software written in C++ version 11.0 and complied in the platform of visual studio 2013. The landmarks were digitized by one trained investigator (TP) and were checked for accuracy by another (HMW).

To quantify reliability of landmark digitization, 60 participants were randomly selected whose digital dental arch models were digitized on four occasions separated by two weeks’ interval. Landmark coordinates digitized on the first and second session were averaged and compared to the average of landmark coordinates obtained from the third and fourth session. Reliability of landmark digitization was determined through the absolute difference between landmark coordinates from the two averages, the Dahlberg’s formula, method of moments, and intraclass correlation coefficient.

### Statistical analyses

Landmark coordinates obtained from the 3D dental arch models provided raw data for geometric morphometric analyses. K-medoids clustering, a non-supervised clustering algorithm with superior robustness to outliers than the conventional k-means clustering^23^, was used to categorize participants’ dental arch into 3 clusters. Analyses were performed separately for dental arch data on age 12, 15, and 18 years. Changes in types of dental arch form from age 12 through 15 to age 18 years were evaluated with Chi-square test. The level of statistical significance was set at 0.05. Analyses were performed in SPSS version 20.0.

Landmarks digitized from the 3D dental arch models were then submitted to Generalized Procrustes Analysis (GPA), which served to remove nonshape information including location, orientation, and centroid size of the configurations. Since the present study aimed to investigate changes in form instead of shape of dental arch, the Procrustes shape coordinates obtained after GAP was then augmented by centroid size through multiplication of Procrustes shape coordinates by centroid size. GPA was performed combining landmark data for all participants at all three age periods so that the resultant coordinates are in the same shape space where valid comparisons of age-related form changes were made possible. Changes in centroid size of dental arch throughout the three age periods were determined by Friedman ANOVA. Post-hoc pairwise comparisons between age 12 and 15 years, age 15 and 18 years, and between age 12 and 18 years were performed using paired t-test. The changes of arch form from age 12 through 15 to 18 years were determined by Procrustes ANOVA, followed by pairwise comparisons among the three age periods under investigation. GPA and further statistical analyses were performed in MATLAB.

## Results

At age 12, 668 children participated in the baseline survey and 627 digital dental arch models were obtained: 17 declined to provide assent to have a dental impression and 24 were excluded because they were undergoing orthotic treatment. At age 15, 435 children participated in the first round of follow-up and 376 digital dental arch models were obtained: 5 declined to provide assent to have a dental impression and 54 were excluded because they were undergoing orthotic treatment. At age 18, there were 383 participated in the second round of follow-up and 352 digital dental arch models were obtained: one declined to provide assent to have a dental impression and 31 were excluded because they were undergoing orthotic treatment. There were 281 participants who participated in all three phases of this study and among them, complete set of digital dental arch models at age 12, 15, and 18 years were available for 225 participants.

The absolute difference between landmarks digitized at the first and second session and landmarks digitized at the third and fourth session ranged from 0.071 to 0.239 mm. Dahlberg’s error values ranged from 0.054 to 0.248 mm. method of moments values ranged from 0.029 to 0.197 mm. ICC values ranged from 0.691 to 0.993.

### Dental arch form clustering and changes in type of dental arch form during adolescence

**Figure 1:**
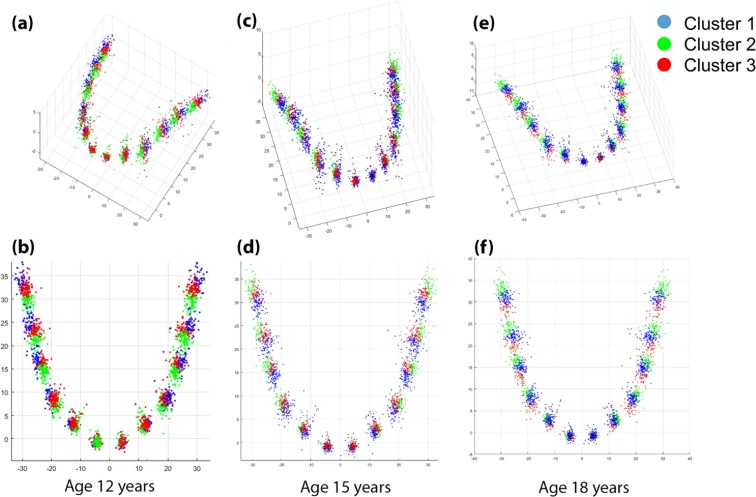
Clustering of maxillary dental arch form age ages 12, 15, and 18 years. 3D view of maxillary dental arch clustering at age 12 years; (**b**) 2D view of maxillary dental arch clustering at age 12 years; (**c**) 3D view of maxillary dental arch clustering at age 15 years; (**d**) 2D view of maxillary dental arch clustering at age 15 years; (**e**) 3D view of maxillary dental arch clustering at age 18 years; (**f**) 2D view of maxillary dental arch clustering at age 18 years.

**Figure 2:**
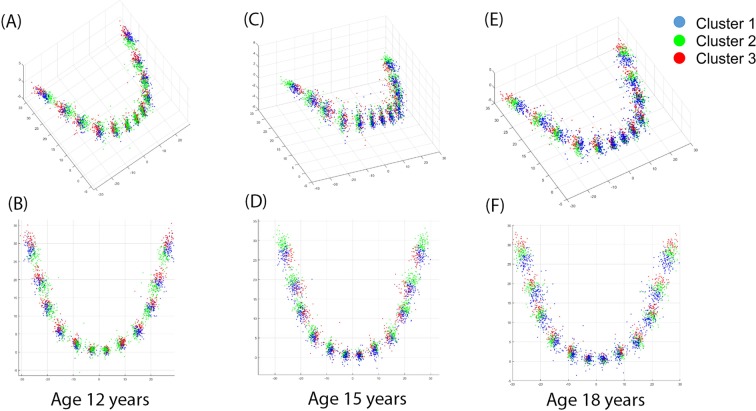
Clustering of mandibular dental arch form age ages 12, 15, and 18 years. (**A**) 3D view of mandibular dental arch clustering at age 12 years; (**B**) 2D view of mandibular dental arch clustering at age 12 years; (**C**) 3D view of mandibular dental arch clustering at age 15 years; (**D**) 2D view of mandibular dental arch clustering at age 15 years; (**E**) 3D view of mandibular dental arch clustering at age 18 years; (**F**) 2D view of mandibular dental arch clustering at age 18 years.

The upper and lower dental arches were clustered into three distinct shape groups: narrow-type, middle-type, and wide-type at age 12, 15 and 18 years (Figs. 1 and 2). There were significant changes in types of dental arch form during adolescence from age 12 through 15 to 18 years for both maxillary (p < 0.001, Table 1) and mandibular (p < 0.001, Table 2) dental arch form.

**Table 1:** Changes in types of maxillary dental arch form from age 12 through 15 to age 18 years.

Age (years)	Types of dental arch form			*p-*value	Pair-wise comparison	
	Narrow N (%)	Middle N (%)	Wide N (%)		Age pair	*p-*value
12	81 (37.2)	88 (4.4)	49 (22.5)	*p* < 0.001***	12 vs 15	*p* = *0.17*^*ns*^
15	92 (42.2)	69 (31.7)	57 (26.1)		15 vs 18	*p* < 0.001***
18	43 (19.7)	101 (46.3)	74 (33.9)		12 vs 18	*p* < 0.001***

^*ns*^*p* > 0.05; ***p < *0.001*.

**Table 2:** Changes in types of mandibular dental arch form from age 12 through 15 to age 18 years.

Age (years)	Clusters			*p-*value	Pair-wise comparison	
	Narrow N (%)	Middle N (%)	Wide N (%)		Age pair	*p-*value
12	81 (27.2)	62 (28,4)	75 (34.4)		12 vs 15	*p* < 0.01***
15	46 (21.6)	82 (37.6)	90 (41.3)	*p* < 0.001***	15 vs 18	*p* < 0.001***
18	90 (41.3)	83 (38.1)	45 (21.6)		12 vs 18	*p* < 0.01**

**p < 0.01; ***p < *0.001*.

Between ages 12 and 15 years, there was no significant change in types of maxillary dental arch form (p > 0.05). In contrast, significant changes in types of mandibular dental arch form was observed from 12 to 15 years (p < 0.001). Between ages 15 and 18 years, there were significant changes in types of both maxillary (p < 0.001) and mandibular (p < 0.001) dental arch form. Between ages 12 and 18 years, significant changes in types of maxillary (p < 0.001) and mandibular (p < 0.001) dental arch form were observed.

### Changes in centroid size of dental arch during adolescence

There were significant changes in centroid size of maxillary dental arch from age 12 through 15 to 18 years (p < 0.001, Table 3). There was a significant decrease in centroid size of maxillary dental arch from age 12 to 15 years (p < 0.001). There was no significant change in centroid size of maxillary dental arch between ages 15 and 18 years (p = 0.15). Significant decrease in centroid size of maxillary dental arch was noted between age 12 and 18 years (p < 0.05).

**Table 3:** Changes in centroid size of dental arch from age 12 through 15 to age 18 years.

	Age (years)	Centroid size	*p-*value	Pair-wise comparison		*p-*value
		Mean ± SD		Age pair	Mean ± SD	
Upper	12	1046.11 ± 49.95		12 vs 15	−9.43 ± 18.59	*p* < 0.001***
	15	1036.67 ± 51.35	*p* < 0.001***	15 vs 18	3.46 ± 35.33	*p* = *0.15*^*ns*^
	18	1040.13 ± 43.84		12 vs 18	−5.98 ± 35.91	*p* < 0.05*
Lower	12	883.46 ± 46.06		12 vs 15	−9.84 ± 15.31	*p* < 0.001***
	15	873.62 ± 48.07	*p* < 0.001***	15 vs 18	1.46 ± 26.26	*p* = *0.41*^*ns*^
	18	875.08 ± 50.80		12 vs 18	−8.38 ± 29.02	*p* < 0.001***

^*ns*^*p* > 0.05; *p < 0.05; ***p < *0.001*.

There were significant changes in centroid size of mandibular dental arch from age 12 through 15 to 18 years (p < 0.001, Table 3). There was a significant decrease in centroid size of mandibular dental arch from age 12 to 15 years (p < 0.001). There was no significant change in centroid size of maxillary dental arch between ages 15 and 18 years (p = 0.41). Significant decrease in centroid size of maxillary dental arch was noted between age 12 and 18 years (p < 0.001).

### Changes of dental arch form during adolescence

Procrustes ANOVA identified significant changes in maxillary dental arch form (p < 0.001, Table 4) during adolescence. There was a significant change in maxillary dental arch form from age 12 to 15 years (p < 0.001). No significant change in maxillary dental arch form was observed between ages 15 and 18 years (p = 0.06). Comparing maxillary dental arch form between age 12 and 18 years, significant form changes were observed (p < 0.001).

**Table 4:** Changes in dental arch from age 12 through 15 to age 18 years.

	Procrustes ANOVA			*p-*value	Pair-wise comparison				*p-*value
	SS	MS	F		Age pair	SS	MS	F	
Maxillary dental arch	1517.73	5.31	1.05	*p* < 0.001***	12 vs 15	632.89	4.43	3.64	*p* < 0.001***
					15 vs 18	379.58	2.65	1.00	*p* = *0.06*^*ns*^
					12 vs 18	1264.12	8.84	6.13	*p* < 0.001***
Mandibular dental arch	2266.02	7.92	4.81	*p* < 0.001***	12 vs 15	881.95	6.17	5.67	*p* < 0.001***
					15 vs 18	448.38	3.14	1.62	*p* = *0.11*^*ns*^
					12 vs 18	1682.99	11.77	6.51	*p* < 0.001***

^*ns*^*p* > 0.05; ***p < *0.001*.

For mandibular dental arch form, Procrustes ANOVA identified significant changes (p < 0.001, Table 4) during adolescence. There was a significant change in mandibular dental arch form form from age 12 to 15 years (p < 0.001). No significant change in mandibular dental arch form was observed between ages 15 and 18 years (p = 0.11). Comparing mandibular dental arch form between age 12 and 18 years, significant form changes were observed (p < 0.001).

## Discussion

The present study investigated types of dental arch based on a large, random sample of Southern Chinese in Hong Kong, examined changes in types of dental arch form during adolescence, and explored changes in centroid size and form of dental arch during adolescence. Although a number of participants were excluded for analyses, we were nevertheless able to collect over 660 3D digital dental model altogether. This uniquely large and population-representative sample, together with the longitudinal nature of our data, increased the power of the present study to identify changes in geometric characteristics of the dental arch and ensured robustness of the study findings.

Arch form is an important aspect to consider in orthodontic treatment planning and in deciding what type of appliances should be employed in the management of malocclusion^24,25^. With advances in technologies and software, assessment of arch forms has become feasible^23^. This approach involves ‘clustering analysis’ to group participants according to dental arch form and investigate changes of dental arch. From digital models of 306 South Korean with mean age of around 20 years (age range: 17–24 years) who had normal occlusion, it was observed that approximately 30% had ‘narrow-type’ maxillary dental arch, 40% had ‘middle-type’ maxillary dental arch, and 30% had ‘wide-type’ maxillary dental arch. For mandibular dental arch, approximately 20% had ‘narrow-type’ arch, 45% had ‘middle-type’ arch, and 35% had ‘wide-type’ arch. Although cross-sectional classification of dental arch form has been performed, longitudinal changes in types of dental arch shape, particularly during adolescence, has not yet been investigated.

Repeatability analyses revealed that there was congruence in landmark digitization across the four sessions, as indicated by the “acceptable”^26^ level of measurements from the four measures of reliability: the absolute difference between landmark coordinates from the first two and last two digitization sessions, the Dahlberg’s formula, the method of moments, and intraclass correlation coefficient. Significant changes in types of maxillary dental arch form were observed during adolescence. At age 12, 37.2% of arch forms were ‘narrow-type’, whereas by age 18, the number decreased to 19.7%. The proportion of ‘wide-type’ arch forms increased from 22.5% at age 12 to 33.9% at age 18. No significant changes in types of maxillary dental arch forms were observed between ages 12 and 15 years. In contrast, significant changes were observed between ages 15 and 18 years.

Significant changes in types of mandibular dental arch form were observed during adolescence. At age 12, 27.2% of arch forms were ‘narrow-type’, whereas by age 18, the number increased to 41.3%. The proportion of ‘wide-type’ arch forms decreased from 34.4% at age 12 to 21.6% at age 18. Significant changes in mandibular dental arch forms were observed between ages 12 and 15 years and between ages 15 and 18 years.

The use of geometric morphometrics to examine arch form has recently emerged in the dental literature^18,19^. Papagiannis *et al*.^19^ examined sexual dimorphism, allometry, age-size and age-shape relationship, and differences between arches in a cross-sectional study based on 133 digital models (age range: 10.6–26.6). No sexual dimorphism was observed in the study. In contrast, shape was significantly associated with arch size albeit the weak strength of association. Age was likewise significantly but weakly associated with arch shape. Shape of maxillary dental arch was found associated with that of the mandibular arch. To date no studies have examined changes in arch form using geometric morphometrics analysis. Geometric morphometric analyses identified significant changes in centroid size and form of both maxillary and mandibular dental arch during adolescence. Significant changes in centroid size and form of dental arch were observed between ages 12 and 18 years for both arches, which were primarily driven by size and form changes between age 12 and 15 years.

The present study examined changes in dentoalveolar features among a large, population-representative Chinese sample that had not received any orthodontic treatment. Dental arch of Chinese has rarely been characterized. A study^27^ evaluated width, length, and height of Chinese dental arch. However, only deciduous dentitions were examined and no age-related changes of dental arch dimensions were investigated. Therefore, their findings were not directly comparable with findings of the present study. For other Asian populations, dental arch dimensions of Vietnamese have been investigated based on a large sample of 4565 12-year-old children^28^. The cross-sectional nature of the study likewise impeded comparison with findings of the present study.

Worldwide, most existing studies on dental arch growth and development have been performed among the white population^29–33^. In white population, a decrease of arch length was observed during adolescence^29^. Bishara *et al*.^30^ reported that dental arch continued to increase in length until age 13 years in the maxilla and 8 years in the mandible. After 13 years, Lundström^31^ and Bishara^32^ found that both maxillary and mandibular arch length decreased with age until around age 30 years. Regarding dental arch width, a continuous increase was observed until 13 years in the maxilla and 12 years in the mandible^10^, which remained stable afterwards^33^. Taken together, findings from the white population suggest a transition towards relatively wider and shorter dental arch during adolescence in both maxillary and mandibular dentitions. Consistent with the decrease in dental arch length observed among the white population during adolescence, we noted significant decrease in size of dental arch among Chinese during adolescence. In addition, consistent with the transition towards wider and shorter dental arch in white population, an increasing proportion of “wide-type” dental arch was observed among Chinese adolescents for the maxillary dental arch. For the mandibular dental arch, however, increasing proportion of “narrow-type” was noted. It should be noted that dental arch width was measured as the distance between canines in earlier studies while^29,33^ while our geometric morphometric approach takes a more holistic view of dental arch shape. Further studies are needed to determine whether the observed difference is due to real ethnic differences or due to methodological variations.

Current studies examining changes of dental arch over time are limited in the number of participants and are potentially biased by being studies of clinical participants rather than of community-based participants. The present study benefits from the power of the revolutionary methods of k-medoid clustering and geometric morphometrics, which provides novel insight into longitudinal form changes of dental arch. In addition, this is the first time dentoalveolar features have been comprehensively evaluated in Southern Chinese.

Several limitations of the present study bear noting. The response rate of the study was less than favorable. This can be attributed to the long period of follow-up (from age 12 to 18 years) and the exclusion of relatively large number of participants who had undergone or were undergoing orthodontic treatment. The relatively high attrition rate may have introduced bias that compromised the generalizability of the study findings to the general population. However, the final sample size was adequate for evaluation of developmental changes in dental arch form, which is substantiated by the statistically significant form changes observed in this study. Post-hoc sample power calculation was not performed for two reasons: first, there is currently a lack of comparable studies based on which to perform the estimation; and second, the study was primarily a proof of concept, i.e., to verify that changes in dentoalveolar features do occur during adolescence.

The present study is of clinical implications. Of note, most changes in the dentoalveolar complex occurred during ages 12 to 15 years as opposed to 15 to 18 years. Age 12–15 years corresponds to a period when most orthodontic treatment is conducted^34^. Thus, the effect of developmental form changes of dental arch during adolescence must be taken into orthodontic treatment planning. Between ages 15 to 18, less frequently were changes in the dentoalveolar complex observed. This would suggest that if treatment is completed at around age 15, the dentoalveolar complex will be relatively stable post orthodontic treatment and chances of relapse due to growth and development is minimal. Nonetheless, some changes in dentoalveolar complex do occur between ages 15 to 18, which still warrants the need for retention after orthodontic treatment. Furthermore, the observed transition towards “wide-type” dental arch in the maxillary dentition and towards “narrow-type” arch in the mandibular dentition provided evidence of the need to adopt differential strategies during orthodontic treatment of maxillary and mandibular dentitions.

In conclusion, our findings suggest most adolescent changes of dental arch take place between ages 12 and 15 years, whereas dental arch form was relatively stable during age 15–18 years.

## Data Availability

The datasets generated during and/or analysed during the current study are available from the corresponding author on reasonable request.
